# Is It Time To Kill the Survival Curve? A Case for Disease Progression Factors in Microbial Pathogenesis and Host Defense Research

**DOI:** 10.1128/mBio.03483-20

**Published:** 2021-02-09

**Authors:** Robert A. Cramer, Caitlin H. Kowalski

**Affiliations:** a Department of Microbiology and Immunology, Geisel School of Medicine at Dartmouth, Hanover, New Hampshire, USA; Duke University Medical Center

**Keywords:** animal models, fungal pathogenesis, fungal virulence, infection site, drug targets

## Abstract

The molecular mechanisms of microbial virulence and host defense are most often studied using animal models and Koch’s molecular postulates. A common rationale for these types of experiments is to identify therapeutic targets based on the assumption that microbial or host factors that confer extreme animal model survival phenotypes represent critical virulence and host defense factors. Yet null mutant strains of microbial (or host) factors often yield extreme survival curve phenotypes because they fail to establish an infection. The lack of infection and disease establishment prevents true assessment of the given factor’s role(s) in disease progression. Here, we posit that the emphasis on extreme survival curve phenotypes in fungal infectious disease models is leading to missed opportunities to identify new fungal and host factors critical for disease progression. We simply do not yet have a sufficient understanding of fungal virulence and host defense mechanisms throughout the temporal course of an infection. We propose that there is a need to develop new approaches and to revisit tried and true methods to define infection site biology beyond the analysis of survival curve phenotypes. To stimulate these new approaches, we propose the (new) terms “disease initiation factor” and “disease progression factor” to distinguish functional roles at distinct temporal stages of an infection and give us targets to foster new discoveries.

## Abstract (precis)

The molecular mechanisms of microbial virulence and host defense are most often studied using animal models and Koch’s molecular postulates. A common rationale for these types of experiments is to identify therapeutic targets based on the assumption that microbial or host factors that confer extreme animal model survival phenotypes represent critical virulence and host defense factors.

## OPINION/HYPOTHESIS

## A TEMPORAL UNDERSTANDING OF FUNGAL DISEASE PROGRESSION: A NEED FOR REFINED TERMINOLOGY?

Human fungal infections cause significant morbidity and mortality in the increasing immunocompromised patient population (1). Rapid advances in many areas of medicine are predicted to only further expand the impact of fungal infections on human health. Moreover, secondary fungal infections have emerged recently as significant complications of critically ill patients with influenza and COVID-19 (2–6). Unfortunately, therapeutic options for human fungal infections remain largely limited to three primary classes of drugs, the polyenes, azoles, and echinocandins (7). The growing crisis of antimicrobial drug resistance further limits available therapeutic options for many human fungal infections. The shear lack of new classes of antifungal agents is rather shocking given the impact these infections have on human well-being. The purpose of this opinion piece is not to discuss the many reasons for this lack of antifungal drug development but, rather, to discuss the underappreciated importance of fungal-mediated disease progression factors and of infection microenvironment biology. We hope this discussion will stimulate new insights into fungal pathogenesis and host immunity mechanisms and impact approaches to novel therapeutic development.

**FIG 1:**
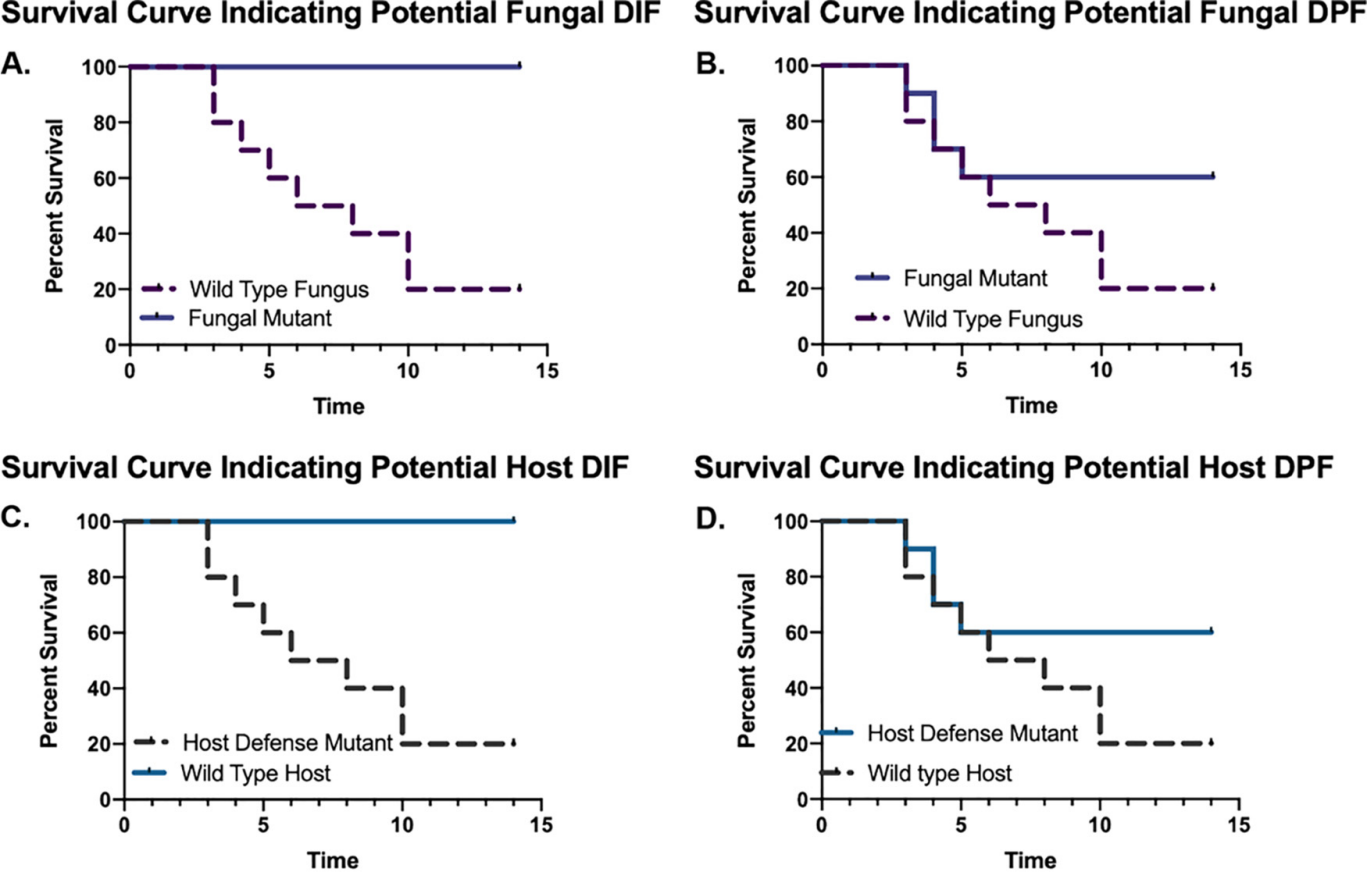
Examples of pathogenesis survival curves. (A) An extreme survival curve result phenotype that has been sought after historically in fungal pathogenesis research to identify virulence factors. Animals challenged with the wild-type strain of the fungus yield significant mortality over the course of the infection, while the mutant strain curve results in limited to no mortality. We suggest these fungal factors be called disease initiation factors (DIFs). (B) An example survival curve where the microbial mutant strain represents a disruption in a microbial disease progression factor (DPF) and is depicted by the separation of the survival curves after infection establishment. (C) An extreme survival curve result phenotype from studies aimed to identify critical host defense factors. Wild-type animals challenged with a given fungus are protected from infection initiation and/or host damage and thus mortality, while loss of the factor results in a severe increase in mortality. (D) When infection is established by a given pathogen and host damage and mortality begin to ensue, some host factors may contribute to disease progression. Loss of these DPF host factors is expected to minimize host damage and mortality as indicated by the increase in host survival in the absence of the host DPF.

Currently, our understanding of fungal disease progression and host immunity is largely limited to fungal and host factors critical for disease initiation. Microbial virulence is studied in the laboratory through many approaches, with Koch’s molecular postulates remaining the gold-standard for identification of new virulence factors, attributes, or mechanisms (8–10). Most often, this gold standard is achieved through the testing of manipulated fungal strains in an appropriate animal model (most often murine) of mycoses. Conversely, on the host side, studies utilize transgenic mouse lines deficient in an immunity related gene challenged with a strain of a given fungal pathogen. For a given fungal or host factor, current thinking requires that loss of the respective factor in a fungal or murine strain results in an extreme survival curve phenotype. Admittedly, there is nuance, and likely debate, in defining what constitutes an extreme survival curve phenotype. However, the most extreme example is a complete or almost complete loss of murine mortality when challenged with a fungal mutant of a given factor, or complete loss of survival of a transgenic mouse line that is challenged with a fungal strain (Fig. 1A and C). Accordingly, these types of extreme survival curve results are celebrated as high-impact virulence or host defense factors. However, high-impact virulence or host factors may have some surprises in store for us when we consider the temporal progression of disease from infection initiation through morbidity and mortality.

While studies identifying fungal virulence and host defense response factors and mechanisms have undoubtedly yielded enormous insights into mycoses, it remains unclear if the majority of fungal virulence and host defense factors identified to date contribute to disease progression beyond the initiation of the infection. Arguably, far fewer factors that are critical for disease progression and/or maintenance of an established infection are currently known. This is in part due to the inherent limitations of our animal models of disease and, namely, our reliance on survival curves as endpoint metrics for defining virulence and host immune resistance factors.

As clinical presentation of most fungal infections involves the treatment of patients with established infections, we must ask ourselves how many of our identified virulence and host defense factors (and the associated concepts surrounding them) are relevant in established infection microenvironments. The many fungal-host interaction complexities that promote disease progression, maintenance, and outcome may never be captured as extreme survival curve results of fungal-host interaction studies. Consequently, the role of many microbial or host factors over the temporal course of the infection is often not fully assessed. For example, what if a fungal mutant is attenuated in pathogenesis so that an infection is never established? It would seem this factor may tell us little about disease progression after infection establishment and, importantly, be a poor therapeutic target. What if a fungal mutant causes host damage through a new mechanism early in the infection that drives animal mortality but whose function is absolutely critical for fungal persistence and disease progression in an established infection microenvironment? This type of factor would tell us more about the infection microenvironment and requirements for fungal persistence but would likely be missed with current approaches. Moreover, what if the virulence factor’s role in infection initiation is contingent on the presence or absence of a specific host factor variable in the population? Survival curve studies often only interrogate one often inbred genotype of a host. Finally, what if the host factor critical for prevention of fungal disease plays no role in host defense, or even a pathological role, following disease establishment? It would seem targeting this host response as a potential therapy would be ineffective at best and harmful at worst. These questions and considerations are important to address and resolve if we are to obtain a fuller understanding of fungal pathogenesis and host defense mechanisms throughout the course of disease progression.

The question becomes how can we better identify and define critical fungal and host factors at distinct time points in disease progression? We propose here that a first step is to develop a new terminology for fungal and host factors that reflects the temporal differences in an infection. Admittedly, virulence and host immunity represent a dynamic spectrum of complex interactions between a host and microbe, and this terminology is thus also likely to be dynamic and subject to debate (much like the term virulence itself!) (11–13). That being said, we feel it is critical moving forward to recognize and experimentally examine the distinction between disease initiation and disease progression after infection establishment. Thus, we propose the terms “disease initiation factor” (DIF) and “disease progression factor” (DPF) to help differentiate between fungal and/or host factors that are critical for impacting disease at temporally distinct stages of the infection.

A DPF is defined as cellular factor, produced by either the microbe or the host, that facilitates microbe persistence, host damage, and continued disease progression in an *established* infection. A DPF, therefore, is not a classical virulence or host resistance factor in the sense that it does not contribute to the establishment or prevention of infection and disease. What constitutes an established infection will vary with each host-pathogen interaction, but we argue that one defining feature is microbe replication and persistence associated with an alteration in host function. In contrast, a DIF is a cellular factor that is essential for the initiation of infection and disease or its prevention. DIFs therefore contribute to subverting host immunity or thwarting microbial pathogenesis at the initiation of the host-microbe interaction. Both DIFs and DPFs have potential therapeutic applicability but in different contexts. For example, a DIF is potentially an excellent prophylaxis target, while a DPF is more suitable as an established infection therapeutic target. In a subset of cases, however, the two may not be mutually exclusive; a DIF may be critical for disease progression later in the course of an infection. Examples of fungal and host DIF and DPF survival curves are presented in Fig. 1.

The general concept that infections are temporally dynamic is, of course, not new but does currently lack specific terminology to clarify the concept and promote research approaches that may yield new insights into pathogenesis, virulence, immunity, and infection outcomes after infection establishment (8, 14). While our focus here is on human fungal infections, the general concept and terminology apply to other infectious diseases, and we provide an illustration of its broad relevance below. It is our hope that this new terminology will foster further discussion around the nuances of infection and disease and, importantly, new research approaches to discover new fungal and host factors critical for disease progression. Accordingly, we suggest that the discovery of DPFs, which are not likely to yield the extreme survival curve results depicted in Fig. 1A and C, but rather the more nuanced survival curves in Fig. 1B and D be celebrated and investigated as rigorously as existing virulence and host defense factors that give extreme survival phenotypes. Next, we discuss some existing examples of DPFs and propose some approaches for their identification and characterization.

## DPF EXAMPLES AND FUTURE DIRECTIONS

**FIG 2:**
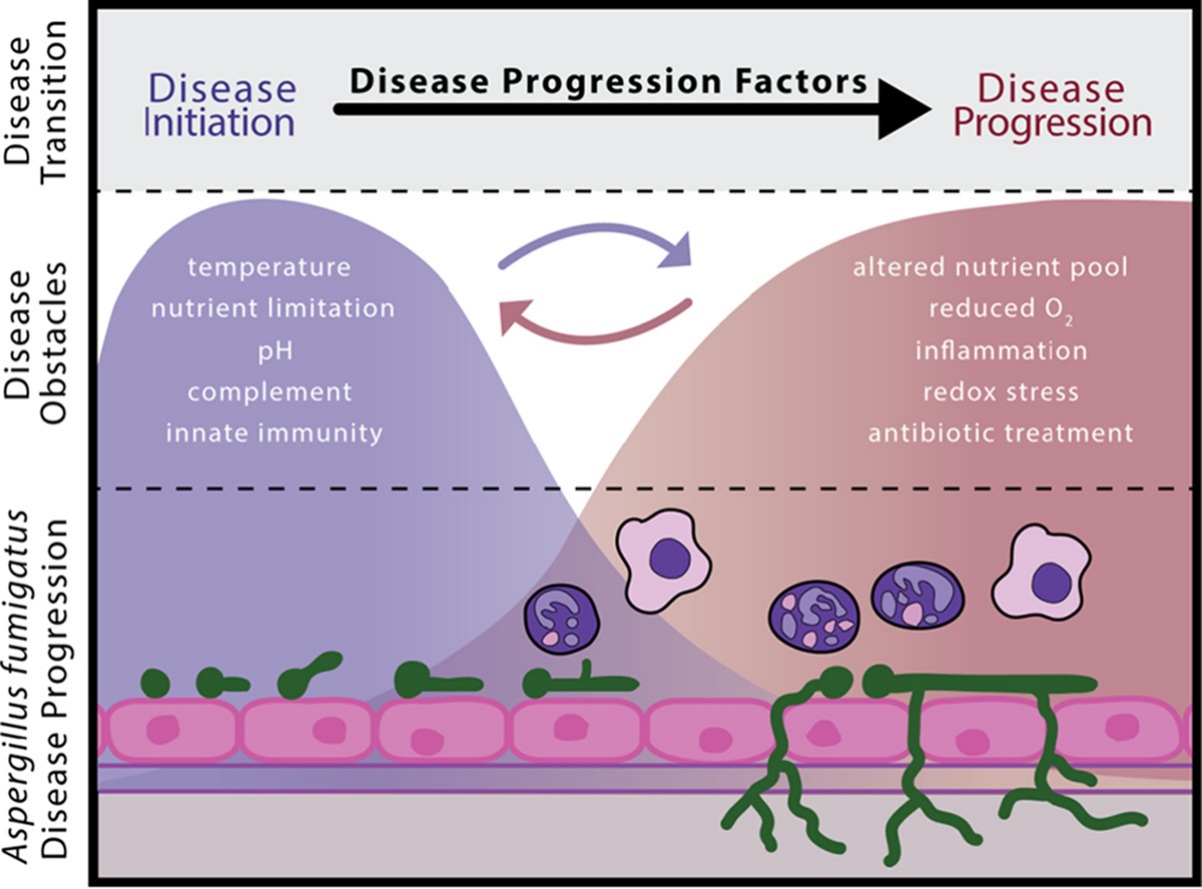
Disease transition from initiation through progression of Aspergillus fumigatus reflects the dynamic nature of infection site pathobiology. Microbes encounter obstacles *in vivo* to which they must adapt to cause and progress disease. These obstacles at the infection site change through the course of infection. For example, Aspergillus fumigatus uses disease initiation factors (DIFs) to establish an infection and the disease invasive aspergillosis. Following adaption to these initial obstacles and disease establishment, A. fumigatus utilizes a second arsenal of disease progression factors (DPFs) to continue to grow, persist, and cause disease. It is after infection and disease establishment that most infections are diagnosed and treated, yet our understanding of infection site pathobiology lags behind our understanding of disease initiation. For many DIFs, it is unclear if they also may function as DPFs, and it is important to recognize that infections represent a continuum of stress conditions that vary temporally and, in some cases, spatially throughout the host when dissemination is part of the infection (circular arrows).

The process of identifying DPFs requires the characterization of temporally distinct stages of an infection that are likely to be variable for each fungus-host interaction. However, patterns of infection can be observed in model systems and across patients and used to identify key signatures to help identify DPFs. Temporal changes in the infection as it progresses are largely dictated by the infection microenvironment and may occur as the fungus disseminates to spatially distinct environments or take place temporally within the same physical infection site. Examples of changes to an infection microenvironment during invasive pulmonary aspergillosis (IPA) are depicted in Fig. 2. Thus, a significant step in DPF identification is to characterize the dynamic nature of the host microenvironment from the initiation of the host-microbe interaction to infection and through its progression. This infection microenvironment characterization, which is not fully known for many infectious disease animal models, would allow for temporal assessment of how microbes and their hosts modulate their physiology to adapt to these changing infection environments.

A fungal example for how spatial changes in the infection microenvironment can alter disease progression is cryptococcosis. In the case of cryptococcosis, infection of the central nervous system (CNS) progresses following an initial pulmonary infection where the yeast disseminates from the lung, across the blood brain barrier, and replicates within the cerebral spinal fluid (CSF). Many of the classic cryptococcal virulence factors, including melanin (15), capsule (16), titan cells (17), and phospholipase B1 (18), are well characterized for their roles in the initial infection of the lung and avoidance of clearance by phagocytes (19). These are clear disease initiation factors for this fungus. However, cryptococcal DPFs would include those factors necessary for progression of disease from the lungs to the CNS, and further factors that facilitate growth within the CSF. Such DPFs have been identified, including fungal urease, which promotes CNS invasion (20), and cryptococcal-produced hyaluronic acid that interacts with CD44 receptors on endothelial cells before facilitating entry into the CNS (21). A recent study has also examined the transcriptome of C. neoformans directly from human CSF, revealing high metabolic activity of the yeast in this environment, and these *in vivo* gene expression data may serve as the basis for identification of additional cryptococcal DPFs (22).

Infections where the microbe occupies a single host niche are less easily separated into distinct phases of disease. In these cases, such as IPA and the bacterial disease tuberculosis when confined to the lung, the stresses that induce microbial DPFs generally occur temporally within the same anatomical site rather than across anatomically distinct sites. For example, during murine IPA, as pulmonary disease progresses, Aspergillus fumigatus forms lesions within the lungs that reach oxygen tensions below ∼1% (23). In addition to alterations in oxygen availability, a switch in available carbohydrate nutrient sources changes during the course of infection (24, 25) (Fig. 2). These are both obstacles following the initiation of infection and disease that A. fumigatus, and other microbes, must respond to and overcome to cause host damage and progress disease. Here, an example of a fungal DPF is the carbon catabolite repressor, CreA, that is not required for IPA infection initiation but, rather, is essential for further disease progression and host mortality. Survival curve studies with a *creA* null mutant yield an interesting, but not extreme, phenotype where mortality is observed similar to the wild-type strain during infection establishment but is reduced as oxygen tensions and nutrient conditions become detrimental to the *creA* null mutant. Thus, survival curves with nuanced results are perhaps a first sign that a given factor may constitute a DPF.

As a nonfungal example to illustrate the broad relevance of the DPF concept, temporal changes in Mycobacterium tuberculosis infection microenvironments are characterized into latent and acute/active stages of infection (26). Several of the microenvironment changes that induce and/or accompany disease progression from latent to active are defined and include a reduction in the vascularization and oxygen tension within the granuloma (26), changes in nutrient utilization by M. tuberculosis (27), and the accumulation of bioactive mycobacterial lipids (28). Notably, a number of mycobacterial factors have been identified that are necessary for development and decay of M. tuberculosis granulomas and are putative DPFs. Examples include mycobacterial isocitrate lyase required for fatty acid utilization (27) and the mycobacterial bioactive lipid trehalose dimycolate, which in association with other lipids induces granuloma decay and promotes active infection (29).

Despite these examples, it remains challenging to identify microbial or host factors involved in disease progression using contemporary approaches. One approach through which new DPFs can be identified *in vivo* is simply by prolonging survival studies in animal models or performing *in vivo* transcriptomics at distinct stages of infection (30). With the former, we need a greater appreciation for studies with microbial or host mutants that demonstrate altered but not extreme survival curve results. These types of modest survival curve phenotypes, too many to illustrate here, likely indicate changes in disease progression worthy of deeper investigation (Fig. 1B and D). One wonders how many potential disease progression factors have been disregarded because mutant studies did not yield high impact survival curve phenotypes. Thus, careful interrogation of the host-microbe interaction is needed with multiple approaches to fully understand and define mechanisms of disease progression.

Additional studies focused on quantitative parameters of disease progression such as microbial burden and markers of host damage can illuminate some of these mechanisms. One powerful approach is simply serial sampling of the infection site for fungal viability through CFU. While this approach is complicated by the filamentous nature of mold pathogens like A. fumigatus and *Mucorales* species, it can be rather precise for unicellular yeast. A similar approach can be used for studies of the host response, for example, by sampling leukocytes from the site of infection and testing for effector cell function in the presence and absence of a given factor at different stages of the infection. However, serial sampling of the infection site is often difficult if not impossible with murine models, though can be highly successful in larger animal models as evidenced by the *Cryptococcus* rabbit model (31). Thus, additional approaches are needed, such as real-time noninvasive imaging of each animal, to monitor disease progression in the presence/absence of factors of interest, and alternative animal models such as the zebrafish that may allow nuanced investigation into conserved aspects of the infection microenvironment (32). As new imaging technologies continue to develop, allowing one to track specific molecules *in vivo*, longitudinal imaging of small animal model infections will be a key tool in defining DPFs.

Another approach for the identification of DPFs is large-scale transposon mutagenesis coupled to high-throughput sequencing (TN-seq) to interrogate essential genes under conditions of interest (33). These methods are also helpful in the characterization of the host microenvironment through the course of infection by using the mutant libraries as biosensors. However, they are limited by a bottleneck at the initiation of infection, where DPFs may also be DIFs. In these cases, DPFs will not be recognized in comparisons at later time points. Modifications such as increased inoculum, reduced complexity of the library, and reduction of the host immune response have all been proposed methods to compensate for this bottleneck *in vivo* (33). The generation of an inducible and robust Tn-seq *in vivo* following the initiation of infection would be ideal for the identification of DPFs. Although methods for inducible fungal transposon mutagenesis exist (34), they have yet to be optimized for use *in vivo*. In addition, inducible systems to drive CRIPSR/Cas9 or Cre-mediated gene editing to generate loss of function alleles at specific time points during disease progression can be used to characterize DPFs once the infection is established. Despite some recent advances with the use of conditional expression systems such as the tetracycline (TET) system, rigorous, timely, and efficient modulation of fungal gene expression *in vivo* should remain a technological goal of the field (35, 36).

Finally, an important recent approach to identify DPFs involves the collection and characterization of within-host isolates of a given microbe via next-generation sequencing (37–40). These studies are increasingly identifying within-host mutations that provide fitness benefits to the microbe and are strong DPF candidates. One seminal example in infectious disease research is the emergence of LasR mutations in Pseudomonas aeruginosa in the cystic fibrosis lung environment (41). These within-host evolution studies also portend the power of *in vitro* experimental evolution approaches to identify DPFs when specific infection microenvironment conditions can be modeled *in vitro* (42).

These longitudinal studies also illustrate another important concept, that the characterization of a microbial or host factor as a DIF or DPF can be context specific and may vary across infection models or microbial strain and host genotypes. In cases where a DPF also serves as a DIF, its characterization should be stated in the context of interest, and a clear opportunity exists to determine whether many classical virulence factors are, in fact, DIFs, DPFs, or both, and how broadly they serve this function in different fungal and host genotypic backgrounds.

## CONCLUSIONS

The use of Koch’s molecular postulates to define mechanisms of microbial virulence largely through the use of animal models has yielded seminal infectious disease discoveries, including in the study of human mycoses. Here, we highlight important considerations for the use of animal models in medical mycology research and propose a new terminology to identify much needed new insights into disease progression mechanisms. Current approaches that rely on animal model survival curve results often fail to address the importance of a given microbial or host factor in an established infection microenvironment. We urge greater consideration for the temporal aspects of disease initiation and progression extended beyond the extreme, high-impact survival curve phenotypes. To facilitate this consideration, we propose specific terms for microbial or host factors that contribute to disease initiation (disease initiation factors [DIFs]) and progression (disease progression factors [DPFs]). These terms will allow for more rigorous application of the damage response framework and encourage deeper characterization of dynamic infection microenvironments (11–13). Mechanistically probing the established infection microenvironment is a challenging task with existing models. However, the microbiology and molecular biology tools available to mycoses researchers continues to expand and will allow for a more precise identification and interrogation of DIFs and DPFs (43). Thus, while survival curve studies remain an important part of our arsenal to identify new pathogenesis and host defense mechanisms and test new therapies, we must continue to expand our investigations into the complex arena of infection site biology. It is highly likely that new discoveries related to established virulence and host defense factors await us in addition to completely novel mechanisms of disease progression that may yield new therapeutic targets and approaches.
